# Patients' and Physicians' Experience With and Acceptability of a Telemedicine Cabin: Mixed Methods Study

**DOI:** 10.2196/55430

**Published:** 2025-04-16

**Authors:** Caroline Villela Galvão de França, Paola Boaro Segalla, Felipe Sebastião de Assis Reis, José Ricardo Silveira Pereira, Alexandre Oliveira de Mattos, Roberta de Moura Ferron, Cleyton Zanardo de Oliveira, Jéssica Bassani Borges, Lilian Quintal Hoffmann, Edmundo Di Giaimo Caboclo

**Affiliations:** 1 Department Occupational Medicine, Hospital BP (A Beneficência Portuguesa de São Paulo), Rua Maestro Cardin, 769, São Paulo, Brazil

**Keywords:** telemedicine cabin, telehealth, teleservice, e-health, connected offices

## Abstract

**Background:**

Telemedicine represents an essential tool with the potential to reduce health costs, thus avoiding patient displacement and improving patient care outcomes, positioning it as a significant social technology.

**Objective:**

This study aims to analyze the implementation of a telehealth cabin at BP Hospital (A Beneficência Portuguesa de São Paulo), focusing on the evaluation of the experiences of both patients and health care professionals, as well as the acceptability of this tool.

**Methods:**

A mixed methods study was conducted with 229 participants, divided into 2 phases. The first phase involved 40 apparently healthy individuals to assess the usability, experience, and satisfaction of this group for the later safe application in the group with clinical complaints. The second phase included 189 participants, with complaints to assess the usability, experience, and satisfaction of patients and doctors. In both phases, participants completed screening questionnaires (to assess the eligibility criteria), a socioeconomic demographic questionnaire before using the cabin, and a questionnaire including the System Usability Scale and the Net Promoter Score (NPS) after using the cabin.

**Results:**

The data analysis of the first phase showed high acceptance of the telehealth cabin, which supported the progression to the second phase. In the second phase, a high usability score was observed among participants with clinical complaints (mean System Usability Scale score of 85.97, SD 15.50) and a high favorability rating (NPS score of 9.4). Health care professionals also reported favorable results, with a usability score of 67.8 and an NPS of 8.0.

**Conclusions:**

The results of this study reinforce the potential for scaling up this practice based on usability outcomes, and highlight its relevance for the development of public policies aimed at expanding access to quality health care in Brazil. This approach improves the interaction of patients with the health care system, while providing professionals with an extended view of clinical conditions through integrated devices, particularly in areas with limited access to medical care.

## Introduction

Health is a fundamental right of the population and must be guaranteed to everyone. It is even included in the Universal Declaration of Human Rights of 1948, in Article 25, which defines that everyone has the right to a standard of living that ensures health, well-being, and health care [1]. Although access to health is often presented as a goal in the health policy, there are several challenges to ensure that right into the Brazilian reality, such as inequalities related to the access and use of health services, discontinuities in the geographical distribution of services, especially of medium and high complexity, in addition to the articulation between health systems of several care levels [2,3].

Among the various proposals developed to improve health care at the national level, the use of technological and telecommunication resources for the exchange of information across different levels of health care, between health professionals, and between doctors and patients, has gained prominence.

According to the Ministry of Health, telehealth uses information and communication technologies to promote the expansion and improvement of medical services. Telemedicine is included in telehealth, related only to remote care through technology. According to the World Health Organization, telemedicine is defined as the “delivery of health services where distance to health professionals is a critical factor, through the use of information and communication technologies to the exchange of valid information for the diagnosis, treatment, and prevention of disease and injuries, research and assessment, and to the continuing education of the providers of healthcare and patients.”

Telehealth has been used in Brazil since the 1990s, emerging in a decentralized and fragmented way in the health sites [4]. Telemedicine has the potential to reduce health costs, avoid patient displacement, and even improve patient care outcomes [5-7]. Although many studies are being published, especially as a consequence of the COVID-19 pandemic, the implementation of telemedicine can still be challenging, mainly due to technological barriers and poor computer knowledge, and even due to resisting change and the patient’s education level [8]. Despite the increasing interest in telehealth, a significant part of the research focuses on the technological aspects rather than on the tool’s adoption and acceptance.

One of the limitations of telemedicine is the lack of possibility to monitor the patient throughout medical care, and some telemedicine platform initiatives have been developed for this purpose. However, today, they provide more complete solutions through a digital health ecosystem, which offers medical instruments to be used by the patient, such as a temperature sensor, stethoscope, dermatoscope, oximeter, otoscope, sphygmomanometer, scale, and height sensor. These new functionalities aim to create resources that can improve even more access and affect the quality of life by bringing doctors and patients closer together.

This research aimed to assess the human aspects of the experience and usability when using telehealth cabins with built-in medical devices, thus expanding the diagnostic and interventional capacity of health professionals in clinical, diagnostic, and interventional capacity practice in its remote format.

To this end, the socioeconomic and demographic profile of the population, the experience and usability of the population in the cabin, the doctor’s experience in providing care through telehealth cabins, and compliance in handling the equipment available in the self-examination cabin were evaluated.

## Methods

### Recruitment

This is a mixed methods study using (1) the System Usability Scale (SUS), (2) the Net Promoter Score (NPS), and (3) a thematic analysis based on participant’ perceptions. The aim was to assess the usability and favorability of health care provided in a connected cabin for symptomatic and asymptomatic employees of the BP Hospital (Beneficência Portuguesa de São Paulo).

### Ethical Considerations

Ethics approval (CAAE: 58070622.9.0000.5483) for this study was provided by the institutional review board of the BP Hospital (Beneficência Portuguesa de São Paulo) on January 31, 2023. All eligible patients signed an informed consent form for this research protocol.

The data was collected on the RedCap platform, which has access control to ensure the security of the data collected in the research. In addition, all data was anonymized to ensure the privacy of the participants.

### Sample Size

The Binomial test was used to calculate the sample size for both parts of the study. For the sampling of asymptomatic individuals (phase 1), an 80% test power was stipulated, a 70% acceptance rate of the cabin under the null hypothesis, and a 90% expected in the sampling rate were specified, resulting in 41 asymptomatic patients.

For the sampling of symptomatic individuals (phase 2), we opted for a conservative scenario. Thus, we set a power of 90%, an acceptance rate of 80% under a null hypothesis, and a 90% expected proportion in the sampling, resulting in 169 patients. Anticipating the possibility of a 12% sample loss, the researchers decided to increase the sampling to 190 symptomatic patients.

### Inclusion and Exclusion Criteria

#### Phase 1: Asymptomatic Patients

The study’s first phase involved 40 apparently healthy individuals with no clinical complaints or decompensated chronic diseases (exclusion criteria for the first phase) to assess the safety and usability of the cabin.

During this phase, all hospital staff received an email with information about the study, inviting asymptomatic individuals to participate. Interested individuals could schedule an appointment by phone to use the cabin.

#### Phase 2: Symptomatic Patients

The second phase included 189 individuals who could be with clinical complaints but did not meet the exclusion criteria, which were: inability to understand and answer the screening questionnaire; patients with signs and clinical symptoms indicating the need for urgent medical care, such as cardiac arrest, shock of any origin, reduced level of consciousness, focal neurological signs, epileptic seizure, chest pain, deep wound, and heavy bleeding.

During this phase, individuals who sought medical care at the institution’s employee health center and who met the inclusion criteria were invited to participate. Those who agreed to participate were enrolled in the study.

### Telemedicine Cabin

**Figure 1.**
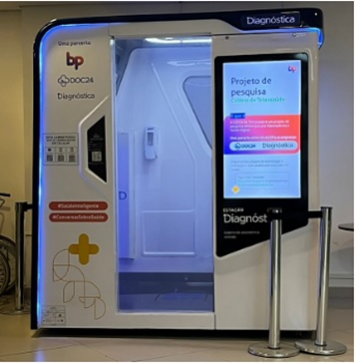
Telemedicine cabin at the location of the research project.

The telemedicine cabin Diagnostica, manufactured in Argentina, was used. It is made of plastic and fiberglass and measures 250×150×230cm dimensions. It has a forced air ventilation and filtering system, environmental lighting with intensity and color control, and an audio and video system for videoconferences (Figure 1).

The participant used his cell phone or the device available in the cabin to receive care and to interact with the person through the app developed for Android and IOS. The patient was accommodated in the cabin and selected the self-examination devices according to their needs.

The following devices were available in the cabin: an otoscope, a digital dermatoscope, and a high-definition camera, all manufactured by Firefly Global in 2021; a stethoscope Riester manufactured in 2021; and an oxyhemoglobin saturation monitor (oximeter), an electrocardiography, a heart rate and temperature monitor, and a sphygmomanometer, models PM6100, all manufactured by Berry, 2021. The patient was instructed to use the app on his cell phone, and the light signals in the cabin were added to a video display to operate the devices for the self-examination.

The cabin recorded the measurements taken by the available devices. The patient had the option, at their discretion, to join the medical consultation at any time during the use of the cabin, even if he did not use the devices. During the synchronous service, the doctor could request that the patient use any of the medical devices to obtain additional clinical information.

Doctors were recruited through direct invitations sent to professionals associated with the hospital. Selection criteria included previous experience with similar technologies and availability to participate in all stages of the study.

Data between the patient and the doctor was transmitted via the Doc24 telehealth platform, implanted in the data processing centers, and connected to the internal service network.

### Study Procedure

Before starting the service, a nursing technician applied the informed consent form. After the participant’s agreement, a screening questionnaire focusing on the inclusion and exclusion criteria and a questionnaire to collect demographic and socioeconomic data before using the cabin were administered. Individuals who met the exclusion criteria or had acute alterations requiring face-to-face assessment were sent to the Employee Health Center for immediate medical care.

The cabin was equipped with a Wi-Fi network and the patient should download an app to access the cabin and the interface (application) which was customized for the project.

During the appointment, the patient, seated in the cabin, can choose between selecting devices for self-examination or opting for a consultation with a doctor. At the patient’s discretion, they have the option to join the medical consultation at any time during the use of the cabin, even without having used the devices.

If the participant chooses to attend a medical consultation during the service, the doctor may ask the patient to use some of the equipment to collect clinical information, or to reuse some of the equipment due to observing poor quality results initially measured by the patient.

Equipment-assisted measurements were available for medical assessment, if required, or later, in the outpatient treatment.

At the end of the service, the research participant was invited to answer the usability questionnaires translated and validated including the SUS, which is a method to measure the usability of several products and services through 10 questions answered on a scale of 1 to 5, where 1 is total disagreement and 5 is total agreement [9], and the NPS, which aims to analyze the patient’s experience and satisfaction [10].

After assigning the NPS score, participants were asked to explain their ratings. Inductive thematic analysis was used to identify a rating [11]. Two authors carefully read each response individually and grouped the responses. Any disagreement was discussed with the first author, and then the response was labeled into 6 categories: “service,” “usability,” “difficulty of use,” “innovation,” “technology resistance,” and “unspecific.” The final step was to group the responses into potential themes.

In order to check the usability of health professionals, the participating doctors also completed the same questionnaires at the end of the daily sessions.

### Statistical Analysis

Study data were collected and managed by an electronic data capture tool, REDCap (Research Electronic Data Capture), and hosted on the BP server [12,13]. REDCap is a secure software web-based platform designed to support data collection for research studies, providing (1) an intuitive interface for the validated data collection, (2) audit trails to track data manipulation and export procedures, (3) automated export procedures for further data downloads to standard statistical packages, and (4) procedures for data integration and interoperability with external sources. Once collected, data were described considering the mean and SD for the numerical variables and the absolute and relative frequencies to the categorical variables. SUS scores were interpreted using the Sauro and Lewis Curved Grading Scale [14].

Patients were classified into 2 groups according to the SUS scale score “Acceptable usability” (SUS ≥68) and “Usability issues” (SUS <68), and the percentage of patients was compared with 70% (for the Symptomatic sample), and 80% (for the Symptomatic sample) by the Binomial test. The cut-off point of 68 was selected because, despite variability in acceptability, this is the threshold at which the technology is considered to have acceptable usability.

The chi-square test or Fisher exact test was used to compare the groups’ qualitative characteristics, and the Mann-Whitney Test was used for the numeric characteristics. The significance level of 0.05 was used throughout the study, and the analyses were performed using SPSS software (version 25; IBM corporation). Graphs were generated using R (version 4.3.2; R studio Team) software and the *ggplot2* package.

## Results

### Overview

The study was divided into 2 phases: the first occurred from March 6, 2023, to March 16, 2023, and 40 asymptomatic patients were included. The second occurred from March 17, 2023, to June 1, 2023, and 189 symptomatic patients were included.

### Assessment of Asymptomatic Patients

The demographic distribution of the asymptomatic patients is shown in Table 1. Most were female, White, married, university-educated, and of higher socioeconomic status.

Concerning the use of the cabin, most of the patients requested support to use the cabin at some point during use. Only 3 patients described the reasons for requesting assistance as difficulties in using the equipment. The appointment had a duration between 10 and 52 minutes.

**Figure 2.**
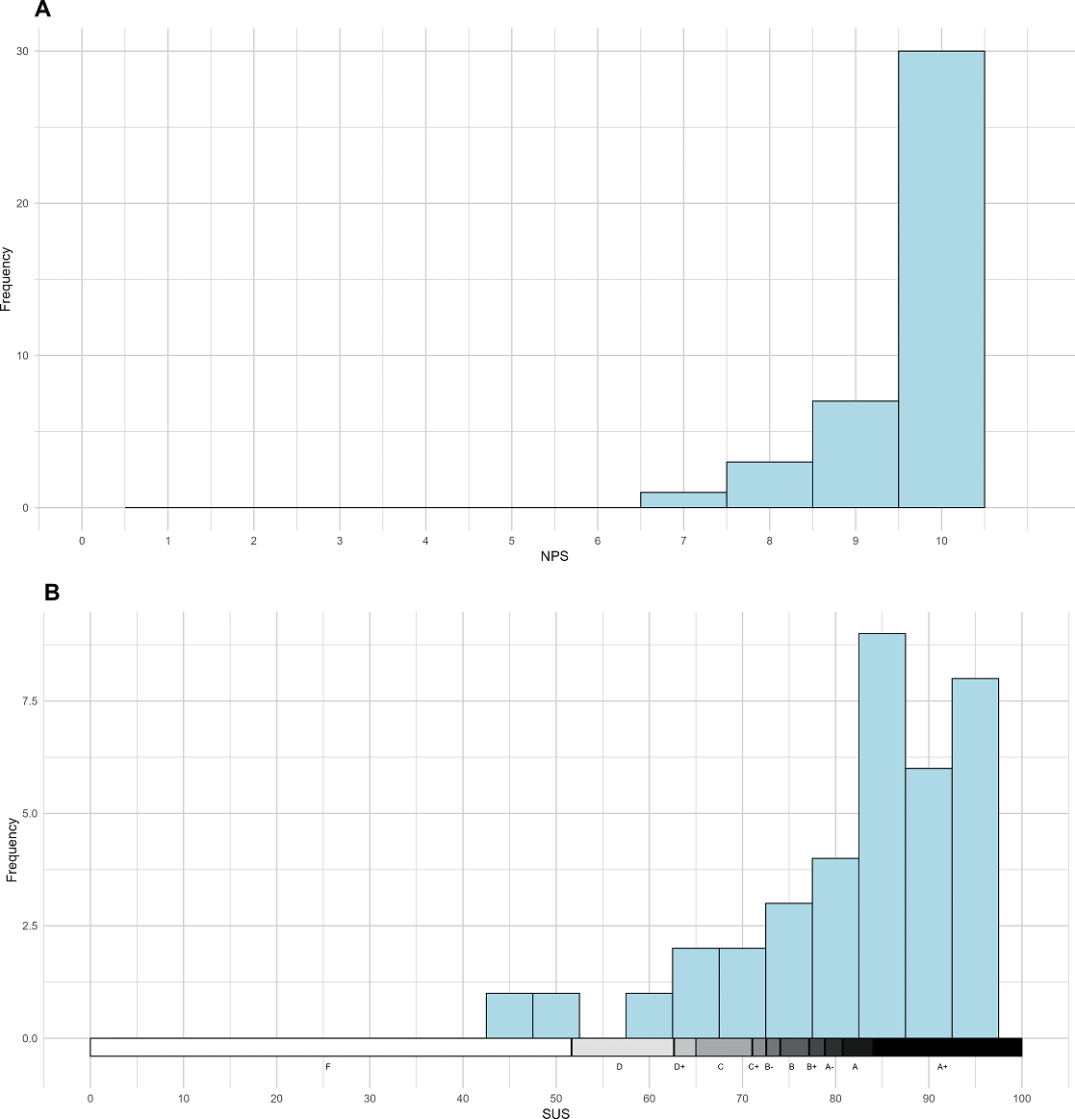
Distribution for asymptomatic patients for the variables: (A) “How likely is it that you would recommend it to a friend or colleague.” (B) Final SUS Score upon graduation using the Curved Grading Scale. NPS: Net Promoter Score; SUS: System Usability Scale.

Upon assessing the satisfaction (Figure 2), we observed that the SUS had a mean value corresponding to Grading A+ (Table 2). When defining the cut-off point for SUS, we identified that only 5 (12.5%) patients had a score of <68 points, whereas 35 (87.5%) had a score of ≥68 points. Thus, the acceptability of 87.5% was statistically different from 70% (*P*=.009) and had a 95% CI 75.5%-100%, justifying the acceptability of the asymptomatic sample and allowing the study to proceed with the sampling of symptomatic patients.

**Table 1.** Distribution of demographic variables for the sampling of asymptomatic patients.

	Values (N=40)	
Sex, n (%)		
Male	15 (38)	
Female	25 (63)	
Ethnicity and race, n (%)		
Asian	2 (5)	
White	24 (60)	
Mixed	8 (20)	
Black	6 (15)	
Education, n (%)		
High school	6 (15)	
College	34 (85)	
Marital status, n (%)		
Single	15 (38)	
Married, common-law marriage, or partnered	24 (60)	
Judicially separated or divorced	1 (3)	
Monthly household income, n (%)		
Up to 3 minimum wages	8 (20)	
4‐6 minimum wages	6 (15)	
7‐11 minimum wages	13 (33)	
Above 11 minimum wages	13 (33)	
Care function, n (%)		
No	36 (90)	
Yes	4 (10)	
Has the patient requested support to use the cabin outside the previously set times?, n (%)		
No	31 (78)	
Yes	9 (23)	
At what point did the patient request medical advice?, n (%)		
Medical advice requested after the tests	24 (60)	
Medical advice requested at the beginning; tests performed upon medical orientation	7 (18)	
Did not request medical orientation	9 (23)	
Age (years), mean (SD)	37.9 (10.9)	
Appointment duration (minutes), mean (SD)	27.0 (10.5)	
NPS^a^, mean (SD)	9.6 (0.7)	
SUS^b^, mean (SD)	84.9 (13.0)	

a NPS: Net Promoter Score.

b SUS: System Usability Scale.

**Table 2.** Distribution of patients by Curved Grading Scale grades of asymptomatic and symptomatic groups.

SUS score range	Grading	Asymptomatic (n=40), n (%)		Symptomatic (n=189), n (%)	
84.1‐100	A+	26 (65)		128 (68.1)	
80.8‐84.0	A	3 (7.5)		8 (4.3)	
78.9‐80.7	A−	1 (2.5)		3 (1.6)	
77.2‐78.8	B+	1 (2.5)		11 (5.9)	
74.1‐77.1	B	2 (5)		8 (4.3)	
72.6‐74.0	B−	0 (0)		0 (0)	
71.1‐72.5	C+	1 (2.5)		2 (1.1)	
65.0‐71.0	C	3 (7.5)		11 (5.9)	
62.7‐64.9	C−	0 (0)		0 (0)	
51.7‐62.6	D	2 (5)		6 (3.2)	
0.0‐51.6	F	1 (2.5)		11 (5.9)	

### Assessment of Symptomatic Patients

The demographic distribution of the symptomatic patients is shown in Table 2. Most were female, white, single, had a college degree, had a household income up to 3 minimum wages, did not perform care functions, and had a mean age was 35.1 years.

Only 5 (2.7%) patients requested support to use the cabin outside the previously established times. The 3 reasons described for requesting support were inconsistencies in performing the cabin and difficulties in using the instruments (otoscope and oximeter) available in the cabin.

**Figure 3.**
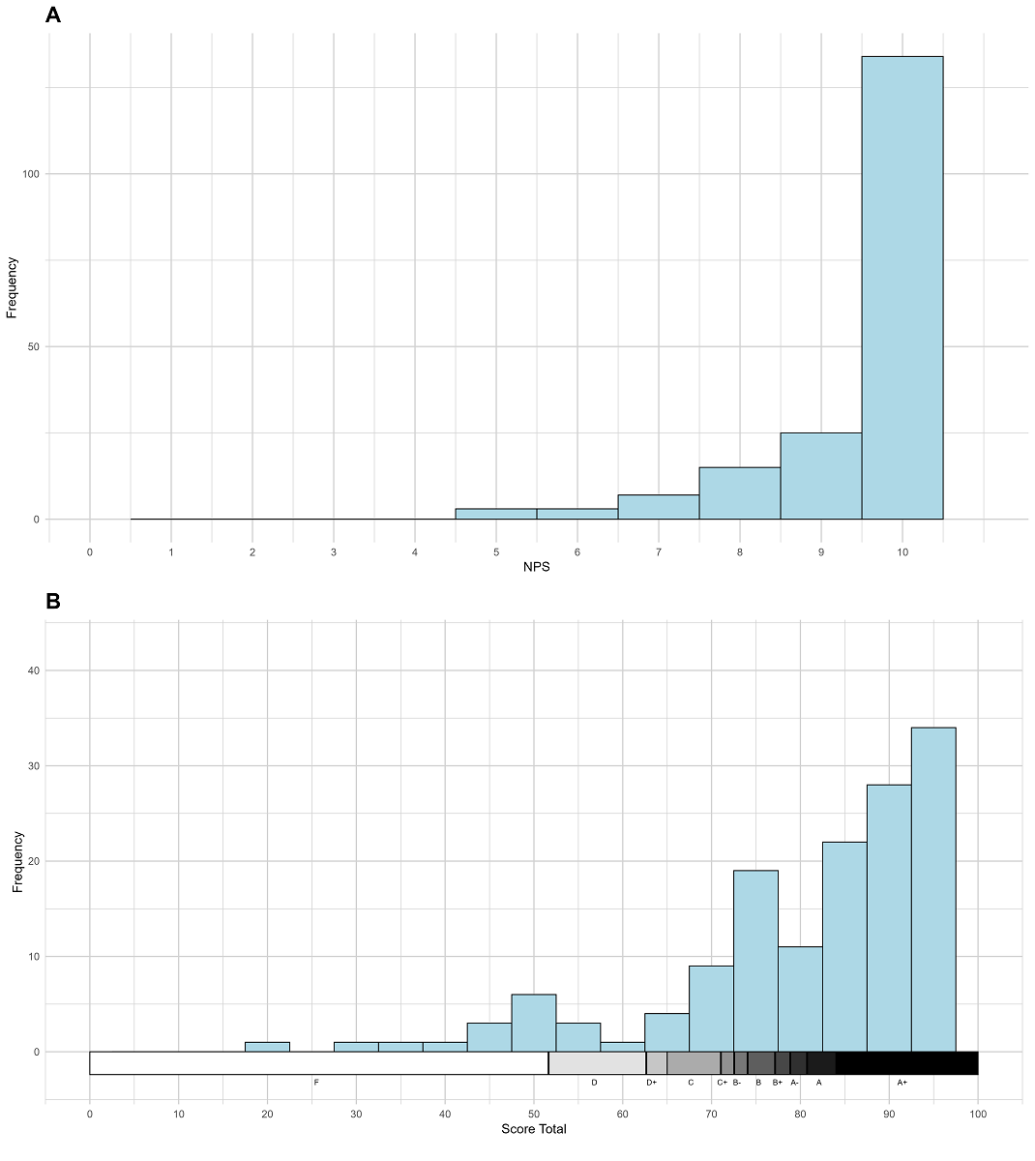
Distribution for asymptomatic patients for the variables: (A) “How likely is it that you would recommend it to a friend or colleague.” (B) Final SUS Score upon graduation using the Curved Grading Scale. NPS: Net Promoter Score; SUS: System Usability Scale.

The most of participants requested medical orientation and performed the measurement under medical orientation. The duration of the appointment ranged from 2 to 58 minutes. Regarding satisfaction (Figure 3), the variable SUS mean corresponds to Grading A+ (Table 2). When assessing the participants’ score in relation to the cut-off point of the SUS scale, we found that only 21 (11.1%) patients had scored <68 points, while 167 (88.8%) had scored ≥68 points. Thus, 88.8% acceptability was statistically different from 80% (*P*=.001) and had a 95% CI 84.3%-100%.

The 21 patients with a score of 68 or less on the SUS were considered in the group “Usability issues” at the cabin. Eleven (52.4%) were female, 13 (61.9%) self-declared White, 11 (52.4%) had a college degree, 13 (61.9%) were single, 5 (45.5%) were married, 7 (63.6%) earned up to 3 minimum wages, 8 (72.7%) did not carry out welfare activities, and had a mean age of 32.64 years (SD 8.43).

Regarding the use of the cabin by these participants, none of them required assistance from the nursing technician, 8 (38.1%) participants requested medical instruction to perform the tests, 7 (33.3%) participants requested medical orientation and made the tests under medical orientation, while 6 (28.6%) participants did not request medical orientation.

The mean appointment duration was 20.38 (SD 10.29) minutes, ranging from 6 to 40 minutes. When comparing the characteristics of patients with SUS ≥68 related to SUS <68, all of them were not statistically significant (attaining a value equal to or higher than *P*=.05) (Table 3), meaning that we did not identify characteristics that could have influenced the difference in the SUS score.

**Table 3.** Distribution of the variables for sampling symptomatic patients and comparison System Usability Scale (SUS) <68 versus ≥68.

		Patient’s number	SUS^a^		*P* value
			<68	≥68	
Sex, n (%)					.05^b^
	Male	55 (29.1)	10 (47.6)	45 (26.9)	
	Female	134 (70.9)	11 (52.4)	122 (73.1)	
Ethnicity and race, n (%)					.99^c^
	Asian	3 (1.6)	0 (0)	3 (1.8)	
	White	113 (59.8)	13 (61.9)	100 (59.9)	
	Mixed	44 (23.3)	5 (23.8)	38 (22.8)	
	Black	29 (15.3)	3 (14.3)	26 (15.6)	
Education, n (%)					.62^c^
	Elementary school	6 (3.2)	1 (4.8)	5 (3)	
	High school	91 (48.1)	9 (42.9)	81 (48.5)	
	College	92 (48.7)	11 (52.4)	81 (48.5)	
Marital status, n (%)					.32^c^
	Single	93 (49.2)	13 (61.9)	80 (47.9)	
	Married, common-law marriage, or partnered	84 (44.4)	6 (28.6)	77 (46.1)	
	Widowed	2 (1.1)	0 (0)	2 (1.2)	
	Judicially separated or divorced	10 (5.3)	2 (9.5)	8 (4.8)	
Monthly household income, n (%)					.88^c^
	Up to 3 minimum wages	95 (50.3)	12 (57.1)	82 (49.1)	
	4‐6 minimum wages	59 (31.2)	5 (23.8)	54 (32.3)	
	7‐11 minimum wages	23 (12.2)	3 (14.3)	20 (12)	
	Above 11 minimum wages	12 (6.3)	1 (4.8)	11 (6.6)	
Care function, n (%)					.59^c^
	No	145 (76.7)	15 (71.4)	129 (77.2)	
	Yes	44 (23.3)	6 (28.6)	38 (22.8)	
Has the patient requested support to use the cabin outside the previously set times^a^?, n (%)					.99^c^
	No	184 (97.3)	21 (100)	161 (97)	
	Yes	5 (2.7)	0 (0)	5 (3)	
At what point did the patient request medical advice?, n (%)					.39^b^
	Medical advice requested after the tests	68 (36)	8 (38.1)	59 (35.3)	
	Medical advice requested at the beginning; tests performed upon medical orientation	85 (45)	7 (33.3)	78 (46.7)	
	Did not request medical orientation	36 (19)	6 (28.6)	30 (18)	
Age (years), mean (SD)		35.1 (10.9)	35.0 (10.2)	35.2 (11.0)	.93
Appointment duration (minutes), mean (SD)		21.8 (11.8)	20.4 (10.3)	22.0 (12.0)	.58
NPS^d^, mean (SD)		9.4 (1.3)	7.7 (2.5)	9.6 (0.8)	<.01
SUS, mean (SD)		86.0 (15.5)	51.2 (11.5)	90.3 (9.1)	<.01

a There was 1 missing value for the variable “SUS” and 1 missing value for the variable “Has the patient requested support to use the cabin outside the previously set times?”.

b Chi-square test.

c Fisher exact test.

d NPS: Net Promoter Score.

Regarding the reasons for their ratings, only 3 participants chose not to answer this question. After careful reading of each response by 2 researchers, the answers were grouped according to the following categories:

- Service: assessment of the interaction between the cabin’s professionals and the patient, considering the attention to the needs, clarification of doubts, and resolution of problems. Examples: (1) “Doctor’s attention and understanding of my problem”; (2) “The speed and attention of the professionals who cared for me”; and (3) “Spectacular service. All my questions were answered. I thought it was excellent.”

- Usability: an assessment of the ease of use of both the interface and the equipment. Examples: (1) “The experience was excellent; I enjoyed doing the tests myself and talking to the doctor about them”; (2) “Super practical, efficient, easy to use the equipment to diagnose, and talk to the doctor afterwards. Loved it!”; and (3) “I found it practical, accessible, and easy to use.”
- Difficulty of use: difficulty in using equipment and tools to locate and understand the functions. Examples: (1) “Difficulty in accurately showing images and pain sites to the professional and the client’s lack of affinity with the equipment can make it difficult to reach a diagnosis”; (2) “Some people may have difficulty with technology”; and (3) “Conflicting instructions between the cell phone and the screen at the self-diagnosis.”
- Innovation: considerations about new products and technologies that can be aggregated to a business or project to bring about improvements. Examples: (1) “Great technology”; (2) “Very advanced technology”; and (3) “For being an innovative, versatile, and practical project.”
- Technology Resistance: resistance to technological change. Example: “In no way does it replace the personal contact with the doctor, as there is no way of knowing for sure what you are feeling.”
- Nonspecific: answers that were not intended or that did not belong to a predetermined group or situation, in this case, to classes of sentences mentioned above. Examples: (1) “Ok”; (2) “I loved it”; and (3) “Top.”

The distributions of the classifications are shown in Table 4.

Most opinions are classified as related to “Usability.” When comparing the classes of sentences of those who scored SUS equal or less than 68 (usability issues) with the remaining (acceptable usability), we found statistical differences in the class “Difficult Usability.” In the group “Usability issues,” 5 (27.8%) scored in this class, while 8 (4.8%) scored in the group “Acceptable usability.”

**Table 4.** Distribution of the classes of sentences.

	Patient’s number, n (%)	System Usability Scale (SUS), n (%)		*P* value
		<68	≥68	
Service				.06^a^
No	106 (57)	14 (77.8)	92 (55.1)	
Yes	80 (43)	4 (22.2)	75 (44.9)	
Usability				.23^a^
No	89 (47.8)	11 (61.1)	77 (46.1)	
Yes	97 (52.2)	7 (38.9)	90 (53.9)	
Difficulty of use				<.01^b^
No	173 (93)	13 (72.2)	159 (95.2)	
Yes	13 (7)	5 (27.8)	8 (4.8)	
Innovation				.99^b^
No	175 (94.1)	17 (94.4)	157 (94)	
Yes	11 (5.9)	1 (5.6)	10 (6)	
Technological resistance				.10^b^
No	185 (99.5)	17 (94.4)	167 (100)	
Yes	1 (0.5)	1 (5.6)	0 (0)	
Nonspecific				.17^b^
No	157 (84.4)	13 (72.2)	143 (85.6)	
Yes	29 (15.6)	5 (27.8)	24 (14.4)	

a Chi-square test.

b Fisher exact test.

### Medical Assessment

At the end of the day’s work, the doctor performing the appointments was oriented to answer the NPS and SUS, evaluating his or her day’s work. The assessment was performed by 2 doctors, reducing the variability of the influence of the medical service on usability; 89% of the services were performed by one of the doctors, and 11% were performed by the other. The distribution of the NPS and SUS is shown in Table 5.

The main difficulties were related to technical difficulties, such as software updates and network connection problems, besides difficulties in adjusting the cabin’s technology (problems with the sensors for measuring the weight and height; problems with the image or screen in the cabin).

**Figure 4.**
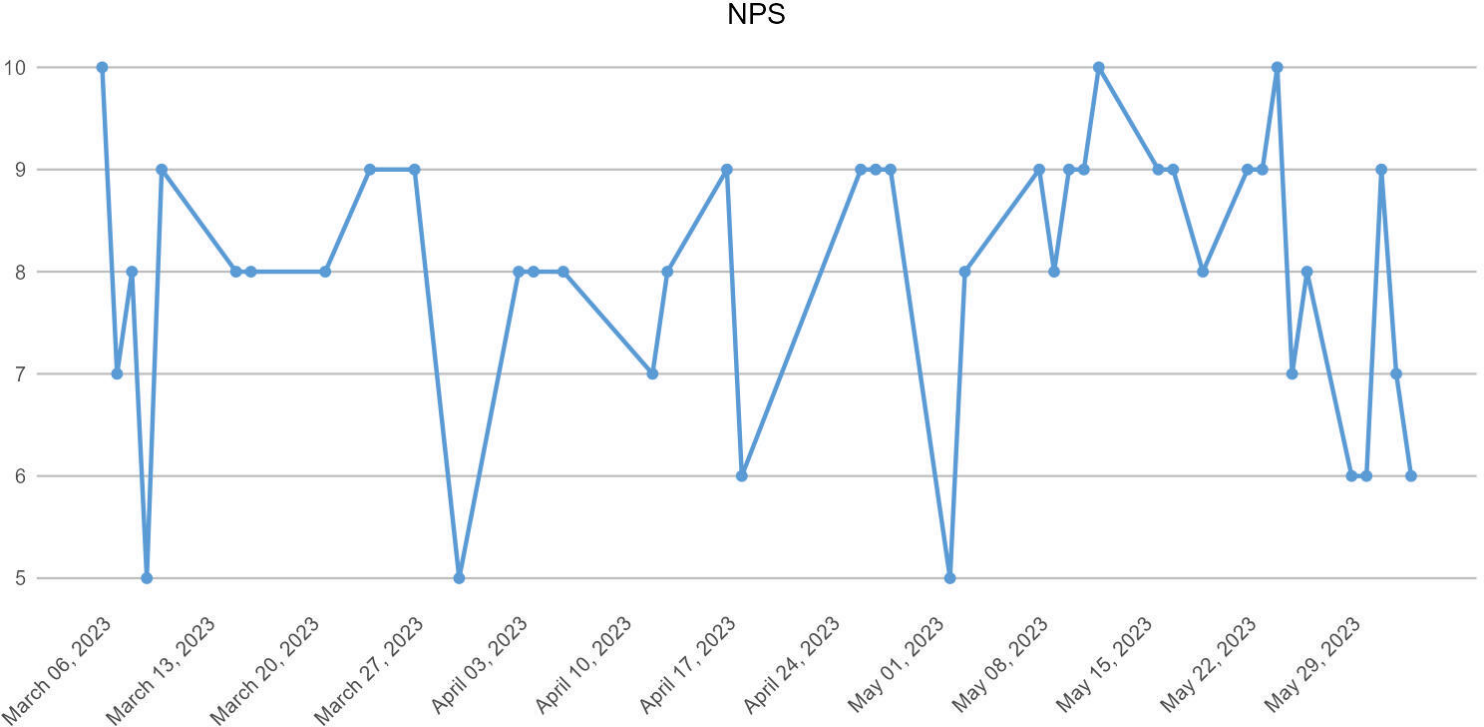
Evolution of the NPS answered by the doctor at the end of each day of service. NPS: Net Promoter Score.

Figures4  5 show the evolution of the allocation of medical grades over time.

**Table 5.** Distribution of the Net Promoter Score (NPS) and System Usability Scale (SUS) values answered by the doctor at the end of each day of service.

	Patient’s number (N)	Mean (SD)		95% CI		Min^a^	1oQ^b^	Medium	3oQ^c^	Max^d^
NPS	41	8.0 (1.4)		7.6-8.4		5.0	7.0	8.0	9.0	10.0
SUS	41	67.8 (13.6)		63.5-72.1		45.0	57.5	65.0	72.5	95.0

a Min: minimum.

b 1oQ: first quartile.

c 3oQ: third quartile.

d Max: maximum.

**Figure 5.**
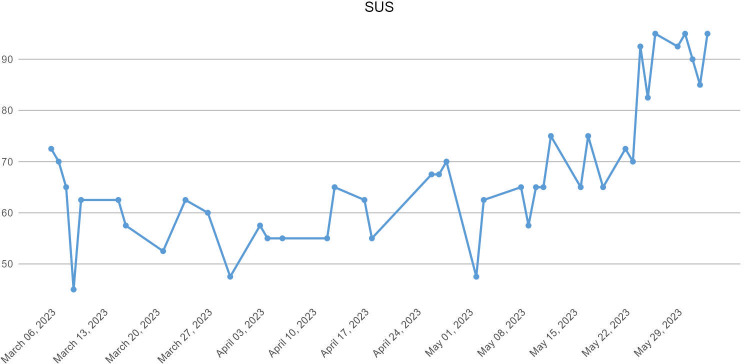
Evolution of the SUS score answered by the doctor at the end of each day of service. SUS: System Usability Scale.

## Discussion

### Principal Findings

The assessment of the implementation of the telehealth cabin in this study aimed to observe the level of favorability and usability of users (doctors and patients) to the cabin and the self-examination devices available, added to their experience of remote service through the technologies available.

The analysis of the results showed that the participants of the first phase of the research (asymptomatic patients) had a different socioeconomic profile than those of the second phase (symptomatic patients), with higher education and monthly income, probably due to the new technology, which attracted leaders and directors of the institution to the research. Although this group was only used to assess the acceptability of both the cabin and the self-examination devices in order to continue the study with symptomatic patients, the outcomes of the favorability and usability were similar between the groups.

Therefore, despite the disparity in access to technology among populations of different economic levels, such a result suggests that the telehealth cabin may have broad applicability for several publics due to the increasing availability and use of mobile devices among the whole population, thus promoting health the equity and reducing social inequalities.

A study with similar equipment performed with French students also concluded that the personal relationship with the technology did not influence the intended use of the telemedicine cabin [15].

A significant portion of the participants did not work in the hospital care sector and therefore did not routinely use the self-examination devices, suggesting that lack of familiarity with the use of the self-examination devices may not impact the use of the cabin. However, this study was performed with professionals working in a hospital, which may bring a certain level of familiarity with those devices, even for those not directly working in the care sector.

Still, we observed that more than half of the patients requested an interview with the doctor, either before or after self-examination, indicating the relevant role of the doctor in such a kind of service.

High favorability and usability were observed for both patient groups (symptomatic and asymptomatic). No statistically significant differences were found when comparing the socioeconomic and cultural profile of those patients with the group of symptomatic who considered the cabin to have acceptable usability, which shows that the usability and favorability of the cabin are unrelated to socioeconomic and cultural factors. However, concerning comments from this group show that some aspects were considered positive even in this group. Furthermore, only one patient described a comment that could be classified in the category “Resistance to Technology,” suggesting that most of the population tested is interested in learning and adopting technologies that improve their routines and experiences.

The findings of this study support the work by Scott Kruse et al [8], asserting that the principal barriers to telemedicine are technology specific. They can be overcome by continuous improvement of these technologies, adequate training of technology users, and personal interaction between the patient and the care provider [8].

Concerning the favorability and usability of doctors who performed the cabin services cabin, we found that the answer was also positive, but with a slightly lower level of usability than that of the patients. This fact seems to agree with other studies that assert that some health professionals may be reluctant. However, this resistance may be related to the difficulty of the technology. Therefore, both the favorability and usability can improve with the advancement of technology and the due training of doctors in web-based physical tests [16,17]. This phenomenon highlights the importance of training programs as a strategy to improve user experience and expedite the adoption of this technology.

A limitation of this study is the participation of only individuals without respiratory symptoms (a consequence of the COVID pandemic) or without symptoms that could indicate a medical emergency. Another limiting factor is the poor accessibility of the cabin for people with disabilities. Furthermore, because the total sampling included people employed at the hospital, and so, in the productive age group and predominantly female [18], along with workers in the day shift, this study may have introduced a bias in the perception of favorability and usability of the participants (for instance, older men are less prone to participate in several telehealth activities [19]).

Finally, we emphasize that the results obtained from health professionals should also be interpreted with caution due to the limited number of professionals who provided care for the study.

It should also be noted that no studies using this type of technology were identified in South America to compare the results obtained. In addition, the fact that this study was conducted after the pandemic may have influenced the results given the impact of the COVID-19 pandemic on people’s lives, which may have affected perceptions.

Based on the favorable assessment of the technology by the participants in this study (patients and doctors) new studies that extend its use to different populations, without any link to the hospital and from different sectors of activity, different work shifts, sex, and different age groups, may bring new inputs to the application of such technology, expanding the service types and favoring the inclusion of the service in remote locations and for diverse populations.

### Conclusions

The authors conclude that the telehealth cabin had good usability and favorability by patients, regardless of the socioeconomic and cultural profile of the population. Doctors who performed the cabin services attested to the cabin’s good usability cabin but with a usability index slightly lower than patients. Most patients used the devices for self-examination, with little need for technical assistance. However, a SUS score greater than 68 does not necessarily mean that there are no usability issues to be addressed.

The outcomes of this research allow us to assess as feasible the expansion of this kind of practice from the point of view of usability and that this study can contribute as a subsidy to the construction of public policies to expand the access of the Brazilian population to qualified medical care, promoting the engagement of patients in their health, providing health professionals with an extended view of the clinical conditions through embedded devices even in areas with a shortage of this type of assistance.

## Abbreviations

BP Hospital: Beneficência Portuguesa de São Paulo
NPS: Net Promoter Score
REDCap: Research Electronic Data Capture
SUS: System Usability Scale
